# Supplementary material for: Towards a “universal translator” for neural dynamics at single-cell, single-spike resolution
Source: ArXiv. 2024 Jul 23:arXiv:2407.14668v2. Preprint. [Version 2] (PMC11302672)
Supplement: Supplement 1 [file NIHPP2407.14668v2-supplement-1.pdf]

A Appendix / Supplemental Material

A.1 Single-session details

Masking scheme ablation. To understand the importance of each masking scheme, we evaluate NDT1 and NDT2 trained with various masking schemes, including temporal, neuron, causal, intra-region, inter-region, as well as the proposed MtM method with and without the prompt token on a selected single session. For both NDT1 and NDT2, Tables 3 and 4 show that the prompted MtM model outperformed the baseline temporal (random token) masking model on most evaluation tasks, except for choice decoding. In addition, each masking scheme performed well on its corresponding task. Figure 6 compares the baseline random token masking NDT2 to the prompted MtM NDT2 across 39 single sessions. The prompted MtM NDT2 consistently outperformed the baseline random token masking NDT2 on all activity reconstruction tasks, while performing similarly on behavior decoding tasks.

Table 3: *The performance of single-session NDT1 trained with various masking schemes on activity reconstruction and behavior decoding tasks.* The metric for activity reconstruction is in units of bits per spike (bps), averaged across all neurons in one session. Behavior decoding is assessed using accuracy for choice and R^2 for whisker motion energy. For all metrics, a higher value indicates better performance.

Masking	Activity Reconstruction				Behavior Decoding	
	Co-Smooth	Forward Pred	Intra-Region	Inter-Region	Choice	Whisker Motion Energy
Temporal (Baseline)	0.84	0.42	-0.20	0.57	0.66	0.56
Neuron	**1.04**	-0.21	-0.22	0.78	0.68	0.60
Causal	0.44	0.48	-0.36	0.23	**0.75**	0.59
Intra-Region	-9.86	-2.97	0.32	-9.06	0.55	0.38
Inter-Region	0.92	0.01	-0.58	**0.90**	0.52	0.59
MtM (Not Prompted)	0.99	0.54	0.42	0.83	0.69	0.61
MtM (Prompted)	0.98	**0.57**	**0.43**	0.84	0.63	**0.61**

Table 4: *The performance of single-session NDT2 trained with various masking schemes on activity reconstruction and behavior decoding tasks.* The metric for activity reconstruction is in units of bits per spike (bps), averaged across all neurons in one session. Behavior decoding is assessed using accuracy for choice and R^2 for whisker motion energy. For all metrics, a higher value indicates better performance.

Masking	Activity Reconstruction				Behavior Decoding	
	Co-Smooth	Forward Pred	Intra-Region	Inter-Region	Choice	Whisker Motion Energy
Random Token (Baseline)	-6.94	0.50	-0.43	-6.95	0.74	0.58
Neuron	0.91	0.18	-0.26	0.62	0.65	0.62
Causal	0.02	0.52	-0.42	-0.20	0.69	0.59
Intra-Region	-10.10	-1.30	0.21	-8.17	0.65	0.43
Inter-Region	0.63	0.18	-0.63	0.66	**0.75**	0.39
MtM (Not Prompted)	0.90	**0.56**	**0.47**	0.80	0.68	**0.62**
MtM (Prompted)	**0.92**	0.54	0.46	**0.81**	0.69	0.62

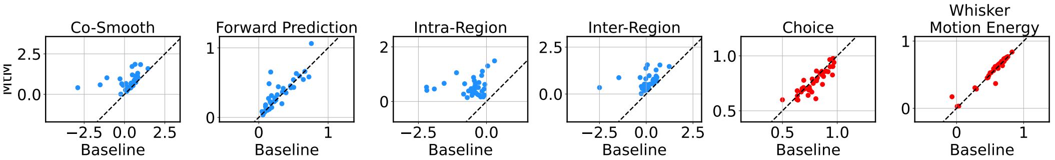

Figure 6: *Comparison of the random token masking baseline and the proposed MtM method for NDT2 on activity reconstruction and behavior decoding across 39 sessions.* For activity reconstruction, each point shows the average bps across all neurons in one session. For choice (whisker motion energy) decoding, each point represents the average accuracy (R^2) across all test trials in one session.

Single neuron evaluation. To better understand neural activity prediction performance at a single-neuron level, we conducted an evaluation of single-session NDT1 on each neuron using bits per spike

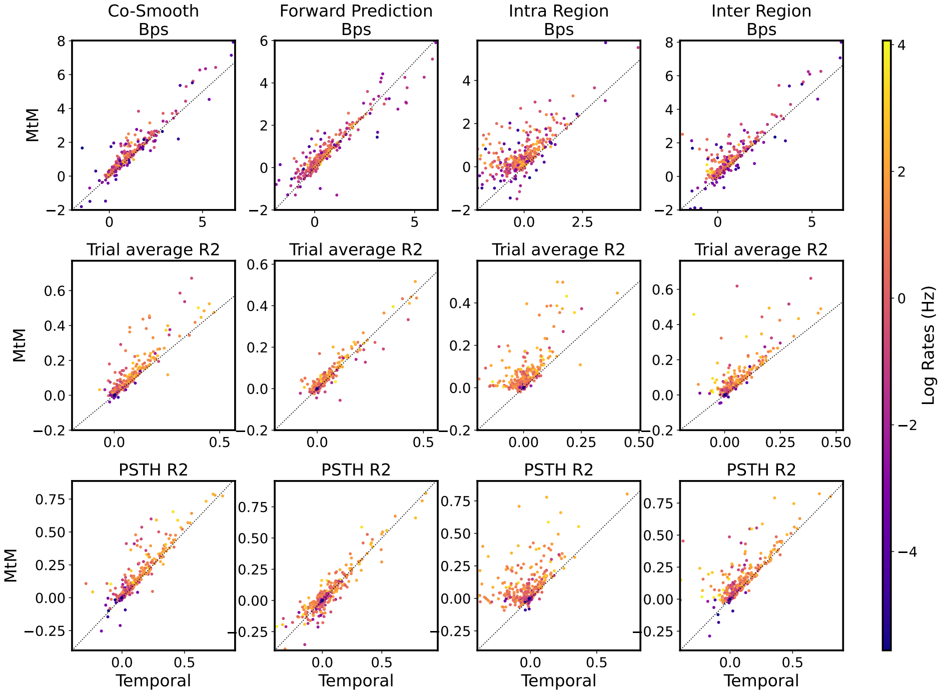

Figure 7: *Single neuron activity reconstruction analysis for NDT1 in one session.* To evaluate the reconstruction quality for each neuron, multiple metrics are computed: Bits per spike (Bps), R^2 between the ground truth and predicted peristimulus time histogram (PSTH R^2), and the single-trial R^2 averaged across all trials (Trial average R^2). Each point represents one neuron, with the color indicating the neuron's log firing rates in Hertz (Hz).

(bps), R^2 between the ground truth and predicted peristimulus time histogram (PSTH R^2), and the single-trial R^2 averaged across all trials (trial average R^2), on one session, in Figure 7. We find that MtM outperforms the temporal baseline across most neurons regardless of firing rate.We did find a strong correlation between the performance evaluated on each metric and the mean firing rates of each single neuron. For the bps (bits per spike) metric, scores for active neurons tend to be more concentrated, while scores for inactive neurons are relatively dispersed, exhibiting both extremely low and high values. For both R^2 metrics, the performance shows a positive correlation with the mean firing rates. In particular, those neurons with extremely low mean firing rates typically exhibited R^2 scores that were extremely low (approaching zero). This observation might be related to the inherent difficulty in predicting the behavior of neurons with low mean firing rates, or the property of metrics themselves. For instance, when applied to neurons with low mean firing rates, the R^2 metric might tend to yield values closer to zero. Across all three metrics (Bps/Trial average R^2/PSTH R^2), our model demonstrated substantial improvements for neurons with relatively higher mean firing rates. However, for neurons with lower mean firing rates, notable improvements were only observed in the bps metric.

A.2 Multi-session details

For NDT2, we report results for pretraining using MtM and the baseline random token masking on 34 sessions of data in Table 5 and Figure 8. In Table 5, we show session-averaged results on the 5 held-out sessions for both the single-session and 34-session pre-trained MtM and random token masking. For both single-session and multi-session, MtM outperforms random token masking across all metrics except choice decoding (where the results are quite similar). Both masking schemes benefit from multi-session pre-training as all unsupervised and supervised metrics improve for the 34-session pretrained models. Similar to the single-session results, the biggest improvements for

Table 5: *Fine-tuning performance of NDT2 pretrained with MtM vs. random token masking on activity reconstruction and behavior decoding.* Activity reconstruction performance is reported in neuron-averaged bps. For behavior decoding, trial-averaged accuracy (R^2) for choice (whisker motion energy) decoding is shown. All metrics are averaged across 5 held-out sessions, and a higher value indicates better performance.

Training	Masking	Activity Reconstruction				Behavior Decoding	
		Co-Smooth	Forward Pred	Intra-Region	Inter-Region	Choice	Whisker Motion Energy
Single-Session	Random Token (Baseline)	-0.87	0.35	-0.99	-0.75	0.84	0.65
	MtM (Prompted)	**0.97**	**0.36**	**0.64**	**0.84**	**0.85**	**0.67**
Multi-Session	Random Token (Baseline)	0.26	0.38	-0.49	-0.39	**0.89**	0.66
	MtM (Prompted)	**1.01**	**0.34**	**0.71**	**0.87**	0.87	**0.67**

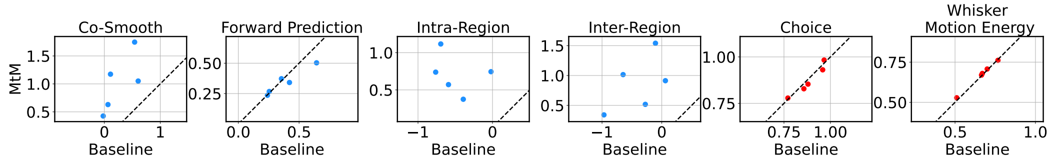

Figure 8: *Fine-tuning performance comparison of NDT2 pretrained with MtM vs. random token masking for activity reconstruction and behavior decoding across 5 held-out sessions.* For activity reconstruction, each point shows the average bps across all neurons in a held-out session. For choice (whisker motion energy) decoding, each point represents the average accuracy (R^2) across all test trials in one session.

MtM are for the unsupervised activity metrics, especially inter- and intra-region prediction. In Figure 8, we show a scatter plot of all metrics for the 5 held-out datasets for MtM and the random token baseline.

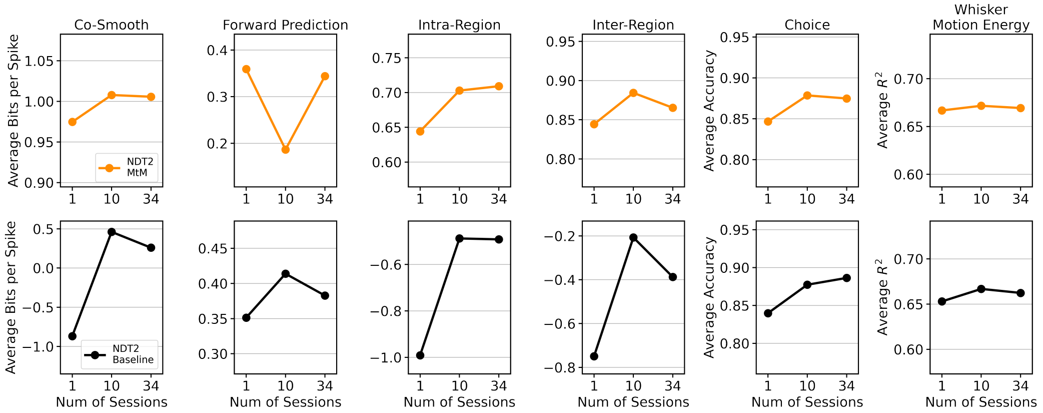

Figure 9: *Comparison of scaling curves between NDT2 pretrained with the MtM method vs. the random token masking baseline.* The reported metrics - neuron-averaged bits per spike, choice decoding accuracy, and whisker motion energy decoding R^2 - are averaged over all 5 held-out sessions used for fine-tuning on both activity reconstruction and behavior decoding tasks. "Num of Sessions" denotes the number of sessions used for pretraining.

Scaling analysis. To examine NDT2's ability of scaling data, Figure 9 shows that NDT2 multi-session pre-training also benefits from scaling from 1 to 10 sessions, but we only observe marginal gains or no improvement going from 10 to 34 sessions. NDT2 (Figure 9) benefits less from multi-session IBL pre-training compared to NDT1-stitch (Figure 4), likely due to the inability of NDT2 with a fixed patch size to handle the large neuron count variations (200 to 1000 neurons) across IBL sessions. Another reason is the NDT2 takes too many neuron patches at the same time, and it's very challenging to deal with this long sequences when data is scaling.

Prompting mode ablation. We also conducted ablation studies on NDT1-stitch of different prompt mode during behavior decoding. We only apply prompt during the model inference, and observe

the different prompt effects to our behavior results. As shown in the Table 6, we ablate four prompt modes.

Table 6: *Evaluation of NDT1's behavior decoding performance when pretrained and fine-tuned using the MtM approach, tested with different prompt tokens at inference time.* Behavior decoding is assessed using trial-averaged accuracy for choice and trial-averaged R^2 for whisker motion energy, with the reported metrics averaged over 5 held-out sessions. For both metrics, a higher value indicates better performance.

Prompting	Behavior Decoding	
	Choice	Whiker Motion Energy
Neuron	0.88	0.68
Causal	**0.89**	0.68
Intra-Region	0.88	0.68
Inter-Region	0.88	0.68

A.3 Training details

We trained our model using AdamW optimizer [22] for 1000 epochs with a learning rate of $1e^{-4}$ using a cosine scheduler. We put a weight decay 0.01 to avoid overfitting. We utilized a relatively small batch size of 16 during the training. We split our dataset based on the session to training, validation, and test set with a proportion of 70%, 10%, and 20%. We saved the model checkpoint based on a trial-average R^2 of top-50 active neurons, which we selected top-50 active neurons and calculated each neuron's R^2 through averaging the trials.

Compute. NDT1-stitch was trained on a machine with a single RTX8000 GPU. NDT2 was trained using Tesla V100 GPUs with 32Gb memory. The 10-session and 34-session NDT1-stitch models were trained for 1 and 3 days, respectively, while the 10-session and 34-session NDT2 models took 2 and 5 days, respectively. Single-session NDT1 and NDT2 models, as well as finetuning, were trained on a single Tesla V100 GPU, requiring 3 to 6 hours. We make sure our experiments are reproducible by seeding.

A.4 Hyperparameters details

The masking ratio is an important model hyperparameter for NDT1 and NDT2. For neuron, intra-region, temporal, and our proposed MtM masking schemes, the masking ratio is fixed at 30%, favoring the performance of the baseline temporal masking method across the activity reconstruction tasks. The causal (next timestep prediction) and inter-region (mask whole region) schemes do not have this hyperparameter, making their performance invariant to the selected mask ratio.

For NDT2, the spatiotemporal patch size is another important hyperparameter. Due to computational constraints, we set it to 128 neurons (≈ 1000 tokens). Future work should analyze how varying the patch size impacts NDT2's performance on neural activity reconstruction and behavior decoding tasks.

Table 7: *Effects of masking ratio on NDT1 performance in neural activity reconstruction.* The reported metrics quantify performance in terms of average bits per spike (bps) across all neurons from a selected session. A higher bps value indicates better performance.

Masking	Mask Ratio = 0.1				Mask Ratio = 0.3				Mask Ratio = 0.6			
	Co-Smooth	Forward Pred	Intra-Region	Inter-Region	Co-Smooth	Forward Pred	Intra-Region	Inter-Region	Co-Smooth	Forward Pred	Intra-Region	Inter-Region
Temporal (Baseline)	0.87	0.73	-0.19	0.44	1.21	0.88	0.31	0.52	0.92	0.88	0.42	0.56
Neuron	1.41	-0.17	0.29	0.55	1.38	-0.08	0.27	0.79	1.25	0.02	0.66	1.10
Causal	0.92	0.82	0.14	0.46	0.92	0.82	0.14	0.46	0.92	0.82	0.14	0.46
Intra-Region	-3.79	-0.76	0.96	-4.06	-3.62	-0.60	0.86	-3.80	-2.18	-0.33	0.43	-2.33
Inter-Region	1.12	0.49	-0.07	1.14	1.12	0.49	-0.07	1.14	1.12	0.49	-0.07	1.14
MtM (Prompted)	1.31	0.79	0.92	1.17	1.24	0.74	0.77	1.08	0.93	0.83	0.71	0.88
